# Oxidative Folding in the Mitochondrial Intermembrane Space in Human Health and Disease

**DOI:** 10.3390/ijms14022916

**Published:** 2013-01-30

**Authors:** Hugo Fraga, Salvador Ventura

**Affiliations:** 1 Department of Biochemistry and Molecular Biology, Autonomous University of Barcelona, Bellaterra E-08193, Spain; 2 Institute of Biotechnology and Biomedicine, Autonomous University of Barcelona, Bellaterra E-08193, Spain

**Keywords:** cysteine motifs, disulfide bonds, Erv1, intermembrane space, Mia40, mitochondria, oxidative protein folding, protein import, oxidative stress

## Abstract

Oxidative folding in the mitochondrial intermembrane space (IMS) is a key cellular event associated with the folding and import of a large and still undetermined number of proteins. This process is catalyzed by an oxidoreductase, Mia40 that is able to recognize substrates with apparently little or no homology. Following substrate oxidation, Mia40 is reduced and must be reoxidized by Erv1/Alr1 that consequently transfers the electrons to the mitochondrial respiratory chain. Although our understanding of the physiological relevance of this process is still limited, an increasing number of pathologies are being associated with the impairment of this pathway; especially because oxidative folding is fundamental for several of the proteins involved in defense against oxidative stress. Here we review these aspects and discuss recent findings suggesting that oxidative folding in the IMS is modulated by the redox state of the cell.

## 1. Introduction

Specific cellular processes cannot be fully understood without considering the multiple pathways that are associated with them. A clear example of this statement is protein translocation into mitochondria [1]. It is known that most mitochondrial proteins (95%) are synthesized in the cytoplasm and imported posttranslationaly to the organelle; however a broad perspective on the way this event is regulated or linked to other cellular processes is missing. This issue is particularly relevant considering that the mitochondria, a key organelle, is associated with an increasing number of human pathologies and that impairment of mitochondrial protein import is lethal [2,3]. Mitochondrial protein import can be modulated by various conditions and, for example in plant cells and in skeletal muscle, the mitochondrial import of proteins has been shown to be affected by the light/dark cycle and contractibility, respectively [4–7]. While several mitochondrial translocation mechanisms are already known [8], among them, a particularly interesting import route is the Mia40 pathway or disulfide relay system, a process that couples the folding, oxidation and import of proteins into the mitochondrial intermembrane space (IMS), since recent evidences indicate that this process is linked to the cell redox state and can indeed be regulated by it.

Here we review and discuss, in light of recent findings, the impact that misregulation of this pathway has on human health.

## 2. The Mia40 Pathway

Recently discovered, the process of oxidative folding in the intermembrane mitochondrial space (IMS) has changed the classic perspective that cysteine oxidation only occurred in the eukaryotic endoplasmatic reticulum or in the bacterial periplasmatic space [9–13]. Several independent studies converged to demonstrate that the oxidoreductase Mia40 (Mitochondrial Intermembrane space Import and Assembly) [14] also denominated Tim40 (Translocase of Inner Mitochondrial Membrane) [15], plays a central role in this process catalyzing the formation of disulfide bonds into target proteins to facilitate both their import and folding into the IMS [12,13,16,17]. So far, several different substrates sharing little homology to each other have been discovered, but it is still not clear how many proteins exploit the Mia40 pathway. Mia40 not only acts as an oxidoreductase but has been shown to possess intrinsic chaperoning activity [18,19]. This latter activity might be relevant for protein import, folding and activity in the IMS, a compartment where the canonical Hsp70 proteins are absent [20,21].

**Figure 1:**
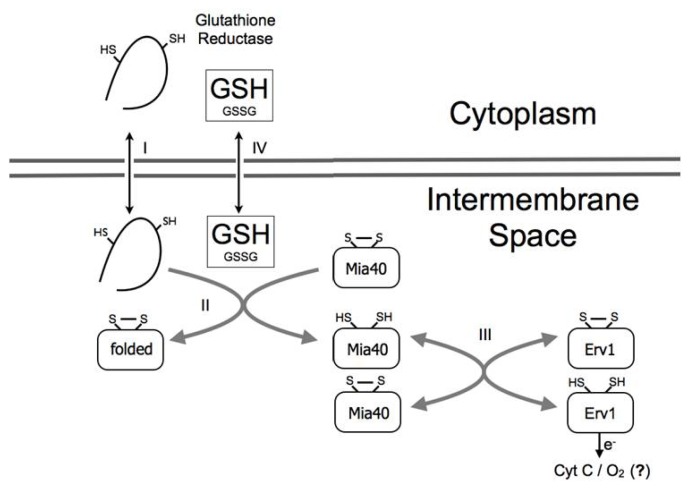
Mia40 pathway is influenced by cytoplasmatic GSH. Reduced and unfolded substrates cross the outer mitochondrial membrane (I) and are recognized by oxidized Mia40 (II). Following the formation of an intermolecular disulfide bond, the substrate is released, oxidized and folded while Mia40 CPC motif is reduced. To allow a new reaction cycle, Mia40 is then reoxidized by Erv1 (III) that can transfer electrons to cytochrome C or directly to oxygen forming H_2_O_2_. GSH has been shown *in vitro* to influence the oxidative folding of several substrates by promoting disulfide reshuffling. Despite contradictory results, the free diffusion of GSH:GSSG from the cytoplasm to the IMS, expected considering the presence of porins, was confirmed recently (IV) [26]. The GSH pool in the cytoplasm also influences the redox state of Mia40 but the molecular mechanisms involved remain unclear.

Disulfide bound formation leads generally to the attainment of the native protein structure. This tight control of the folding reaction by disulfide bond cross-linking is proposed to be the driving force for vectorial translocation of proteins across the outer membrane to the IMS without any requirement for an additional input of energy [20,22]. Data obtained from *in vivo* and *in vitro* experiments supports an oxidation mechanism involving the initial formation of an intermediate displaying a mixed disulfide between Mia40 CPC redox site and one of the substrate cysteines [23], followed by a nucleophile attack of a second inner cysteine of the substrate to the Mia40-substrate intermediate, releasing the reduced catalyst and the substrate with the first native disulfide bond formed (Figure 1). The transfer of electrons implies, however, that the catalyser must recycle to its original redox state. Mia40 is not an oxidase, it is unable to transfer its electrons to molecular oxygen and to guarantee its participation in a new reaction cycle it is reoxidized by the FAD-linked sulfhydryl oxidase Erv1 (Essential for Respiration and Vegetative grow) that transfers the electrons to cytochrome c, which finally delivers the electrons to the respiratory chain [24]. Alternatively, Erv1 can also transfer the electrons to molecular oxygen producing hydrogen peroxide [25]. It is still not clear how/if these two different electron transfer reactions are regulated, since yeast deletion mutants without functional respiratory chain complexes III and IV do not exhibit any change in the Mia40 redox state at least in oxygen concentrations above 20% [26]. An alternative reaction mechanism where Erv1 is involved directly in substrate oxidation through the formation of a transient ternary complex Erv1:Mia40:substrate has been recently suggested [27,28]. Reflecting the relevance of this process for the cell, Mia40 and Erv1 are essential proteins in yeast and mutations in Erv1 human homologue, Alr (see section 4), result in deficient respiratory complex function [29,30].

## 3. A Link between the Mia40 Pathway and the Redox State of the Cell

Mitochondria constitute the major source of reactive oxygen species within a cell; therefore it is likely that oxidative stress can itself influence the redox state of Mia40 and the correct import of several proteins. Substrates for the Mia40 pathway include proteins relevant to Cox biogenesis (Cox12, Cox17, Cox19, Cox23), several small Tim (Translocase of inner membrane) chaperones and enzymes involved in anti-oxidant defense, such as Ccs1, and consequently defects in this pathway are likely to result in multiple effects due to ineffective protein import impacting several mitochondrial processes. Moreover, a recent report has demonstrated that Mia40 also assists in the import of Tim22 [31], the main subunit of the TIM22 translocase, responsible for mediating the integration of carrier preproteins into the inner membrane [32].

In principle, and irrespective of the identity of the catalyser, Mia40 or a Mia40-Erv1 complex, the electron transfer from the substrate to Mia40 and Erv1 should be sensitive and likewise perturb the cell redox state as Mia40 CPC redox site must be oxidized to be functional [26]. Indeed, the first report of a functional link between Mia40 oxidation state and the respiratory chain was demonstrated by Bihlmaier *et al.*, when it was observed that in the absence of oxidized cytochrome c, Mia40 is shifted to its reduced state [24]. Moreover, while previous reports, based on the use of redox sensitive GFP sensors, have proposed that the IMS constitutes a redox environment independent from the cytosol, a recent report by Kojer *et al.* show that the IMS and the cytosol are, as could be expected from the presence of porins, able to freely exchange glutathione (GSH). Using an assay based on recovery following oxidative challenge with the oxidant diamide followed by subsequent washout it was observed that both the IMS and cytoplasm displayed similar recovery kinetics and that both were dependent on the NADH-dependent enzyme glutathione reductase (Glr1) present in the cytoplasm [26,33]. Although the reason for the apparently contradictory results between groups apart from the use of different fluorescent probes is still unclear, in favor of the idea that the cytoplasm can influence the redox state of the IMS and therefore of its proteic components, Kojer *et al.* demonstrated that, in yeast, the Mia40 redox state is influenced by the deletion of Glr1 [26]. This is a very interesting finding specially because GSH has been shown to increase the oxidative folding rate of Mia40 substrates *in vitro* and to facilitate protein import into mitochondria, presumably by promoting the reshuffling of disulfides towards the native connectivity [18,34]. Although Kojer and coworkers did not observe differences in the import of canonical twin CXnC motif Mia40 substrates in Grl1 mutants, it was previously shown that induction of oxidative stress by the administration of paraquat to isolated mitochondria resulted in the impairment of mitochondrial protein import of the ornithine transcarbamylase precursor [35]. In the same line of argument, mitochondria protein import in plants was shown to be sensitive to the redox status of sulfhydryl groups on the outer surface of the inner membrane [36].

While conclusive data on a possible redox control mechanism for IMS Mia40 catalyzed protein oxidation are still missing, it is apparent considering the above mentioned data that this process cannot occur as an isolated non-regulated event. In fact, similar oxidative folding processes have been shown to be tightly regulated at the endoplasmatic reticulum and it is likely that the same occurs at the mitochondria.

An important class of enzymes dependent on the Mia40 pathway are superoxide dismutases (Sods) that catalyze the dismutation of superoxide anions to hydrogen peroxide and oxygen. These ubiquitous enzymes have great physiological significance and therapeutic potential, being involved in diseases frequently associated with ageing, such as familial amyotrophic lateral sclerosis (FALS), Parkinson’s disease and several other neurological disorders. Eukaryotes possess two intracellular Sods, a copper- and zinc-containing enzyme (Sod1) representing 90% of the total Sod activity and located primarily in the cytosol (12) and a manganese-containing (Sod2) enzyme in the mitochondrial matrix. These two enzyme forms also coexist in mitochondria as Sod1 is present in the IMS [37]. The import of Sod1 into the IMS depends indirectly on the Mia40 pathway [38].

**Figure 2:**
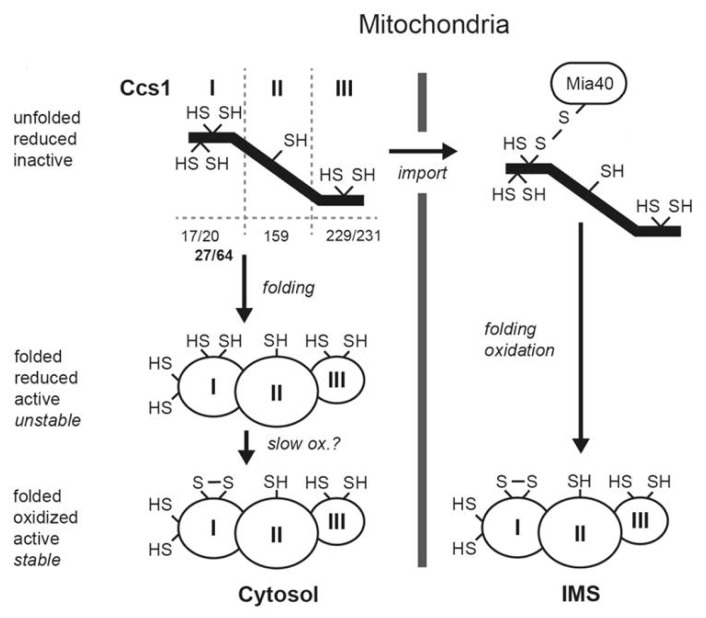
CCS1 and Sod1 import into IMS depend on Mia40. Ccs1 and Sod1 coexist in the cytoplasm and IMS space. Because Ccs1 and Sod1 do not contain mitochondrial targeting signals, their cellular partition is controlled by a folding trap mechanism involving the oxidation of the Ccs1 domain I Cys 27 and 64 residues by Mia40 present in the IMS (S. cerevisiae numbering, in bold) [39]. Cys 27 and 64 are not essential for activity as the protein is fully active in its reduced state present in the cytoplasm. Recognition by Mia40 can also occur independently of cysteine residues as proposed to occur for human Ccs1 where those residues are not conserved [40]. Figure adapted from [39] with authors permission.

Indeed, for its maturation in yeast, Sod1 requires the copper chaperone for Sod1 (Ccs1), which promotes the formation of the disulfide bond and the incorporation of the copper ion in the dismutase [37,41]. Ccs1 is a multidomain protein consisting of three domains containing conserved cysteine residues [42–44] (Figure 2). The amino-terminal domain I of 74 residues of Ccs1 in S cerevisiae harbors a CxxC motif and shares structural homology with the copper chaperone Atx1 that has the ability to bind copper ions. It also contains two other cysteine residues at positions 27 and 64. Domain II, ranging from residue 79 to 223, has homology to Sod1 and mediates docking between Ccs1 and its substrate through heterodimerization [42,45,46]. Domain III comprises the *C*-terminal 26 residues harboring a CxC motif that is essential for the activation of Sod1 [40–42]. Recent studies have identified cysteines at positions 27 and 64 in domain I of Ccs1 as critical for mitochondrial import and interaction with Mia40 (Figure 2). Upon Ccs1 interaction with Mia40, these cysteines form a structural disulfide bond that stabilizes the overall fold of domain I [39,47]. Curiously, although the cysteines are essential for the accumulation of functional Ccs1 in mitochondria, they are dispensable for the enzymatic activity of cytosolic Ccs1. It is important to note, that even though Sod1 and Ccs1 coexist in the cytoplasm and mitochondrion they do not contain mitochondrial targeting sequences. Therefore, it has been proposed that the Mia40-mediated oxidative folding of domain acts a switch, controlling the cellular distribution of Ccs1 and, consequently, of active Sod1 (Figure 2). Several experiments suggest that indeed this folding-driven modulation occurs *in vivo*. For example, the expression of Ccs1 with an *N*-terminal mitochondrial targeting sequence in a Ccs1 deletion background leads to its localization to the IMS in both yeast and mouse brain providing an extra protection again oxidative damage [48–50]. In addition it is known that mammalian tissue culture accumulates higher amounts of Sod1 and Ccs1 in the mitochondrion under hypoxia [51] as a result of the increase in Ccs1 import [51]. According to Kawamata *et al.*, the accumulation of active Sod1 in the IMS in hypoxia could constitute a preventive defense against the burst of reactive oxygen species that are formed upon reoxygenation due to accumulated substrates in the respiratory chain [51]. How oxygen concentration is coupled to Mia40-Erv1 activity is still to be discerned, but a recent work by Yang *et al.* has showed that overexpression of the human Mia40 homologue, CHCHD4, resulted in an increase in oxygen mitochondrial consumption rate [52]. In the same work, CHCHD4 was shown to be involved in the regulation of the HIF pathway, a key mechanism in the cell response to molecular oxygen concentration [52]. The HIF pathway deregulation is associated with human cancer, and CHCHD4 knockdowns displayed reduced tumorogenesis, opening perpectives for drug development based on CHCHD4 modulation [52,53].

## 4. Genetic Diseases Associated with Mia40 Pathway

Because mitochondria are essential for energy production in eukaryotes, errors in its metabolism are normally associated with pathological states. Indeed, one of the challenges in the field of mitochondrial genetics is to identify the mechanisms that can explain diseases that result from mutations in housekeeping genes. The same applies for the disorders that might result from mutations on substrates of the Mia40 pathway. The first example of this possibility was the identification of the human deafness dystonia syndrome or Mohr-Tranebjaerg syndrome (MTS/DFN-1), a recessive, X-linked neurodegenerative disorder characterized by progressive sensorineural deafness, cortical blindness, dystonia, dysphagia and paranoia that is caused by mutations or deletion of the DDFP-TIMM8a, the human homologue of yeast Tim8 [54,55]. The small Tim proteins belong to an evolutionarily conserved protein family composed by Tim8, Tim9, Tim10, Tim12 and Tim13 that associate to form different functional complexes. One of these assemblies, the 70 kDa, (Tim9)3(Tim10)3 complex is essential for escorting a class of multimembrane-spanning proteins across the IMS prior to their insertion into the mitochondrial inner membrane [30,56]. In other complex, Tim8 partners with Tim13 into the IMS to form a complex that facilitates the import of Tim23, a key component of the mitochondrial preprotein translocase [57,58], but also a group of Ca^2+^-binding aspartate/glutamate carriers [59]. Tim protein members can only form functional complexes in their oxidized state with two stable intramolecular disulfide bonds [60]. As occurs with all Tim family proteins, Tim8 is a substrate for Mia40, containing a twin CX3C motif. The importance of Mia40 oxidation for the folding of the Tim family is stressed by the report of a patient with a missense mutation C66W where a cysteine of the CX3C motif is mutated to a tryptophan [54]. This single mutation results in the failure of Tim8 to be recognized and oxidized by Mia40 and consequently aborts its ability to form complexes with Tim13 with deleterious effects for health [54].

Another clinical case linked to the Mia40 pathway is the recent description of a mutation in the Alr; the above mentioned human homologue of Erv1 [29,61]. The fact that Alr mutations result in pathology is not surprising since previous *in vitro* reports have shown that mutations in Erv1 resulted in several deleterious phenotypes in yeast that included disrupted mitochondrial morphology and dysfunction and loss of mtDNA [62,63]. This specific mutation was first found in an inbred Moroccan family with three siblings affected by congenital cataract, progressive muscular hypotonia, sensorineural hearing loss, and developmental delay. Linkage analysis, followed by the sequencing of candidate genes, revealed the presence of a missense mutation (R194H) in Alr and, as expected, this mutation affected IMS protein import. Alr can exist in two different isoforms, the long form exists predominantly in the IMS and contains an 80 aa *N*-terminal extension relative to the shorter form, sfAlr, an extracellular cytokine involved in intracellular redox-dependent signaling pathways [52]. Arl is a homodimer, and R194 is located at the interface, close to the intersubunit disulfide bridges and the arginine guanidino moiety participates in three H-bonds: with two main-chain carbonyl oxygen atoms (from R194 itself, and from C95 of the intersubunit disulfide of the other protomer) and with the 2′ OH of the FAD ribose. While the R194H mutation has minimal effect on the enzyme activity it affects the conformational stability of Alr decreasing the melting temperature, increasing the rate of dissociation of FAD from the holoenzyme, and strongly enhancing the susceptibility to proteolysis and to reduction of its intersubunit disulfide bridges by glutathione; therefore likely reducing the complement of active enzyme in the mitochondrion. Using a yeast strain where both Mia40 and Erv1 were replaced by its human homologues, Sztolsztener *et al.* demonstrated that although the AlrR194H mutant displayed similar steady state levels of Mia40 and Alr compared to the WT protein at permissive temperature, those values were lower when the temperature was shifted to 37 C [64]. Moreover the interaction Mia40:Erv1 was apparently weaker when the mutation was present [64].

The above cases represent “mild” affections where mutations affect the proper functioning of the pathway without impairing it completely. Indeed, due to its fundamental role on IMS protein import, it is unlikely that mutations totally blocking Mia40 pathway could produce viable organisms. However, it is very possible that several others disorders resulting from failures in the folding and import associated to IMS are still concealed by the rather incomplete actual knowledge of the substrates transiting this pathway. Even with the respective gene isolated, deafness dystonia syndrome, was only associated with mitochondrial import when the function of the TIM proteins was discerned. It is clear that only with a complete understanding of the targeting and import process can we attempt to manipulate the system to achieve desirable outcomes and possible therapeutics.

## 5. Conclusions

The central role of Mia40 in the process of oxidative folding in the IMS is now widely recognized. However, while a significant amount of information has already been obtained on the mechanism using model substrates, its regulation and links to the remaining cellular processes still need to be clearly discerned. As mentioned in the above text, several exciting results now suggest that oxidative folding at the IMS is controlled by the redox state of the cell and, in fact, this is not totally surprising. The links between the cell redox state and Mia40 pathway are multiple and the IMS is rich in processes that may influence the local glutathione pool, for example the subunits of the respiratory chain, and antioxidant enzymes. Moreover, Erv1 and Alr can produce hydrogen peroxide and superoxide anion and the existence of a feedback inhibition, such as the one present in the ER, is a possibility to be explored.

While the details of Mia40 regulation may remain for the moment unclear, its relevance for human health is obvious considering its pivotal role in protein import to mitochondrion. Key enzymes, such as Ccs1 and Sod1, depend on Mia40 activity being retained in the IMS and it is likely that as the number of identified substrates increases, a number of associated pathologies will be discerned in parallel. This is particularly relevant if we consider that the number of possible targets of Mia40 was considerably expanded with the report that the enzyme can act on substrates other than the canonical twin CXnX motif proteins. Only with full knowledge of these substrates in hand can we perhaps understand the relevance of this pathway for mitochondrial function and human health. The recent report that associates Huntington’s disease phenotype to Mia40 pathway disruption is clear evidence of this statement [65].
